# Frequency and Pattern of MRI Diffusion Restrictions after Diagnostic Catheter Neuroangiography

**DOI:** 10.3390/tomography9030082

**Published:** 2023-05-12

**Authors:** Elisabeth Kesseler, Svenja Tafelmeier, Omid Nikoubashman, Anca-Maria Iancu, João Pinho, Martin Wiesmann

**Affiliations:** 1 Department of Diagnostic and Interventional Neuroradiology, University Hospital RWTH Aachen, 52074 Aachen, Germany; ekesseler@ukaachen.de (E.K.); stafelmeier@ukaachen.de (S.T.); onikoubashman@ukaachen.de (O.N.); aiancu@ukaachen.de (A.-M.I.); 2 Department of Neurology, University Hospital RWTH Aachen, 52074 Aachen, Germany; jferreiradep@ukaachen.de

**Keywords:** angiography, diffusion-weighted imaging, infarction

## Abstract

(1) Background: We investigated the frequency, location, and lesion size of diffusion restrictions (DR) in magnetic resonance imaging (MRI) of asymptomatic patients after diagnostic angiography and assessed risk factors for their occurrence. (2) Methods: We analyzed diffusion-weighted images (DWI) of 344 patients undergoing diagnostic angiographies in a neuroradiologic center. Only asymptomatic patients who received a magnetic resonance imaging (MRI) examination within seven days after the angiography were included. (3) Results: Asymptomatic infarcts on DWI were identified in 17% of the cases after diagnostic angiography. In these 59 patients, a total of 167 lesions were noted. The diameter of the lesions was 1–5 mm in 128 lesions, and 5–10 mm in 39 cases. Dot-shaped diffusion restrictions were found most frequently (*n* = 163, 97.6%). None of the patients had neurological deficits during or after angiography. Significant correlations were found between the occurrence of lesions and patient age (*p* < 0.001), history of atherosclerosis (*p* = 0.014), cerebral infarction (*p* = 0.026), or coronary heart disease/heart attack (*p* = 0.027); and the amount of contrast medium used (*p* = 0.047) and fluoroscopy time (*p* = 0.033). (4) Conclusions: With an incidence of 17%, we observed a comparatively high risk for asymptomatic cerebral ischemia after diagnostic neuroangiography. Further measures to reduce the risk of silent embolic infarcts and improve the safety of neuroangiography are warranted.

## 1. Introduction

Noninvasive imaging techniques, such as computed tomography angiography (CTA) or magnetic resonance angiography (MRA), allow for the diagnosis of neurovascular disease in many cases [1]. However, digital subtraction angiography (DSA) is still the method of choice for certain indications, such as the exclusion of small aneurysms or the evaluation of arteriovenous malformations [1,2,3]. Being invasive, DSA carries a certain risk of symptomatic cerebral infarction with an estimated frequency of 0.3–2.6% [4,5,6,7]. Moreover, silent embolic infarctions without the appearance of neurological symptoms occur, which can only be detected using diffusion-weighted magnetic resonance imaging (DWI) [8]. In a widely cited study published in 1999, Bendszus et al., reported such asymptomatic DWI lesions following DSA in 23% of cases [8]. Measures such as heparinization and air filters have been proven to reduce the rates of asymptomatic DWI lesions [9]. Nevertheless, a risk for the occurrence of clinically silent embolic infarctions remains. We hypothesized that the stringent use of continuously flushed catheters, the heparinization of flush solutions, and advances in angiographic materials might have reduced the frequency of DWI lesions following diagnostic angiographies.

Our aim was, therefore, to assess the frequency, location, and lesion size of diffusion restrictions (DR) in magnetic resonance imaging (MRI) studies of asymptomatic patients after diagnostic angiography, as well as to find the risk factors for their occurrence.

## 2. Materials and Methods

Patients: From January 2010 to May 2020, 2340 diagnostic angiographies (DSA) were performed in our department. From these, we retrospectively analyzed the data of all 344 consecutive patients who were asymptomatic for neurological symptoms after the angiography and underwent an MRI within seven days after the DSA. Craniocervical and spinal angiographies were included in the study. Patients who had undergone an interventional angiography procedure between the time of the diagnostic angiography and the MRI were excluded from the study. As reported in a separate analysis [manuscript submitted for publication], neurological complications occurred in 16 of the 2340 angiographies (0.68%), of which only 3 patients suffered from permanent neurologic symptoms (0.13%). These angiographies were excluded from the present study.

Cerebral angiography: Examinations were routinely performed on a biplanar digital subtraction angiography (DSA) system (Artis Zee, Siemens, Erlangen, Germany). Before and after the DSA, all patients underwent neurological examinations performed by the Department of Neurology of our institution. Blood pressure, ECG, and O_2_ saturation were monitored during the procedures. In most cases, a 4F or 5F sheath was used via a right-sided transfemoral approach. A continuous flushing system with NaCl 0.9% (1000 mL NaCl 0.9% pressure irrigation, Fresenius) and 1000 IU heparin per 1000 mL NaCl was connected to the catheters for all examinations. Routinely, neither air filters nor anticoagulation were used before or during angiography. Predominantly, catheters with Vertebral (V1) or Simmons (SIM2) tip configurations (Cordis, Santa Clara, CA, USA) and hydrophilic guidewires (Terumo 35 Standard, Terumo, Shibuya, Japan) were used. Manually injected non-ionic contrast medium was used in all cases (Solutrast 300, Bracco Imaging, Konstanz, Germany). After the examination, the puncture site was manually compressed for 10 min and dressed with a compression bandage.

Angiographies were always performed by a team consisting of an experienced neuroradiologist and a neuroradiologist in training. They were assisted by a radiographer. Since our institution is a tertiary neurovascular referral center, the angiographies included varied considerably from single-vessel supraaortal studies to complex diagnostic studies, including super selective catheterizations or bilateral injections. The medical indication, fluoroscopy time, and the amount of contrast medium used were listed in the intervention protocol. Each patient was kept under inpatient care for at least one night after the examination. Any change in the neurological status was documented.

Data analysis: After obtaining approval from the Ethics board of our institution, we listed and analyzed the following data: age, sex, date of angiography, medical indications for angiography, and pre-existing vascular risk factors. Other factors recorded were as follows: reported difficulty of catheterization, emergency conditions, amount of contrast medium injected, number of catheters used, angiography time (weekdays 8 a.m.–5 p.m. or off-duty), medications administered during and after angiography, sheath size, puncture site, probing time, fluoroscopy time, radiation exposure, and number and name of vessels probed.

MRI: 151 MRI examinations were performed using a 1.5 T MRI scanner (Magnetom Aera, Siemens, Erlangen, Germany) and 193 examinations were performed using a 3 T MRI scanner (Prisma, Siemens, Erlangen, Germany). Every MRI examination was performed with a standardized protocol including fluid-attenuated inversion recovery (FLAIR), T2*-weighted gradient-echo (T2*), and a diffusion-weighted echo-planar imaging sequence (DWI). Using apparent diffusion coefficient (ADC) maps, it was possible to correct false-positive findings on the DWI images due to a T2-shine-through effect. MRI imaging for all patients included in the present study was performed within the first 7 days following angiography [mean: 2.1 days]. Hyperintense signal abnormalities on DWI images were categorized for lesion size (1–5 mm vs. larger than 5 mm), lesion shape (dot-shaped or wedge-shaped), and location (territorial, cortical, or white matter). We also assessed whether diffusion restrictions (DR) were found in areas that were supplied by arteries probed during the angiography or in non-probed areas.

Statistical analyses: Statistical analysis was performed with SPSS 27 (IBM Statistics 27.0.1.0 Chicago, IL, USA). After testing for normal data distribution with Kolmorogow tests, t-tests were applied. If data did not meet the requirements of normal distribution, chi-square tests or Mann–Whitney U tests were used as applicable. All tests were two-sided. A *p* value < 0.05 was considered statistically significant.

## 3. Results

Of the total 2340 DSA examinations performed in our department between January 2010 and May 2020, 344 patients underwent MRI within 7 days after the DSA without having symptoms suggestive of ischemic complications and were included in our analysis. Our cohort comprised 336 craniocervical and 8 spinal angiographies.

We found asymptomatic DR in 59 of the 344 patients (17%); all of them had undergone craniocervical angiography before. Following spinal angiography, no DWI lesions were found. Demographic characteristics, indications for angiography, and risk factors are detailed in Table 1. Details of the DSA procedures are presented in Table 2.

Predisposing risk factors significantly correlated with an increased risk of DWI lesions comprised the following: higher age, and history of atherosclerosis, cerebral infarction, or chronic heart disease (CHD). Characteristics of the angiography procedure significantly correlated with an increased risk of DWI lesions comprised the following: increased fluoroscopy time and increased amount of injected contrast medium (Table 1 and Table 2).

**Figure 1:**
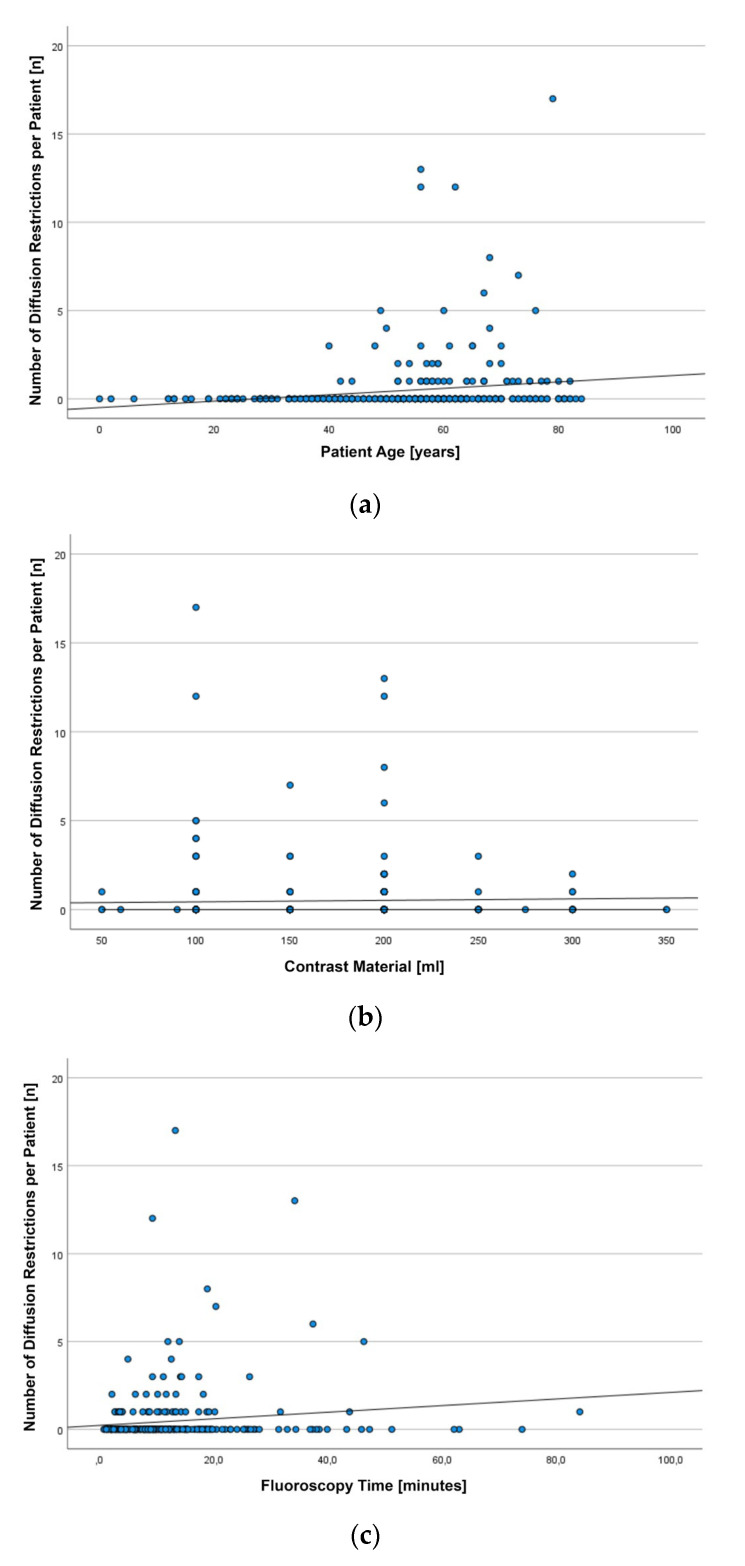
Scatterplots depicting the number of diffusion restrictions (DR) observed on diffusion-weighted images (DWI) after diagnostic angiographies in 344 patients with regard to patient age (**a**), the volume of injected contrast material (**b**), and fluoroscopy time (**c**).

In total, we found 167 DR in the 59 patients harboring DWI lesions. On average, 2.83 ± 3.36 DR were noted per patient (median: 1; IQR: 2; range: 1–17). The number of DR per patient was significantly correlated with patient age (r = 0.251, *p* < 0.001), volume of injected contrast material (r = 0.107, *p* = 0.049), and fluoroscopy time (r = 0.130, *p* = 0.020). However, the observed correlations were weak (Figure 1).

**Figure 2:**
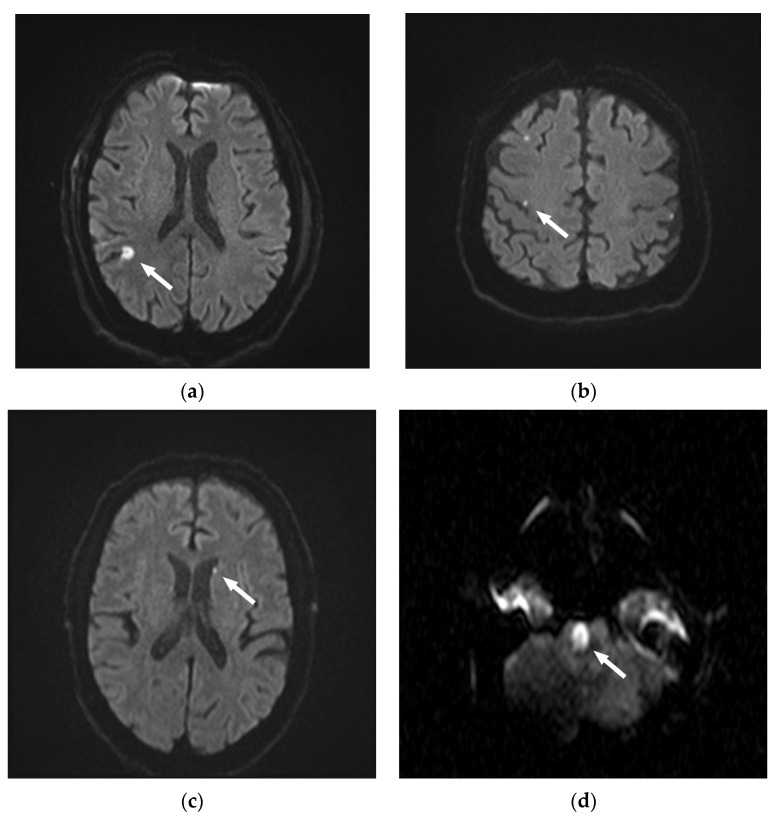
Illustrative figures of the types of diffusion restrictions (DR) observed on diffusion-weighted images (DWI) after diagnostic angiographies. (**a**) 75-year-old patient with a wedge-shaped DR > 5 mm, cortical. (**b**) 79-year-old patient with three dot-shaped DR 1–5 mm. Two cortical DR on the right side, and one DR in a non-probed area on the left side. (**c**) 56-year-old patient with one dot-shaped DR 1–5 mm in the left-sided white matter. (**d**) 40-year-old patient with a wedge-shaped pontine DR.

None of the patients harboring DWI lesions showed neurological deficits during or after the examination. In total, 128 lesions were between 1–5 mm in size, while 39 were larger than 5 mm. The largest lesion had an extension of 10 mm. Four territorial diffusion restrictions were found, 55 were located in the white matter, and 104 were located cortically. Only in 4 patients, lesions were found in areas of non-probed vessels, whereas 55 patients displayed DR only in areas that were supplied by arteries probed during angiography. Dot-shaped embolic diffusion restrictions were found most frequently (*n* = 163, 97.6%). Only four DR were presented as wedge-shaped (Figure 2).

## 4. Discussion

A wide range of incidence rates (0–26%) of asymptomatic DWI lesions has been reported in the literature [3,8,9,10,11]. Kato et al. described an incidence of 26% in 13/50 patients [3]. Of these 50 patients, only 41 patients had received diagnostic angiography, and of the 41 patients who received angiography, 8 suffered from new cerebral ischemia. Consequently, the incidence of DWI lesions after diagnostic procedures would have amounted to ‘only’ 19.5%. The remaining nine patients, however, received interventional angiographies, a fact that contributed to the high incidence rate [3]. In a frequently cited study, Bendszus et al. found a total incidence rate of 23% (23/100) [8]. His study sample comprised 66 diagnostic and 34 interventional procedures. DWI lesions were found in 17 diagnostics and 6 interventional angiographies. Considering only diagnostic procedures, the incidence of DWI lesions would have amounted to 17%. In a follow-up study, Bendszus et al. included 150 diagnostic angiographies, of which 50 procedures were performed using air filters between catheters and syringes, 50 procedures were performed with heparin administered as a bolus and continuous infusion, and 50 procedures were performed as control group without the use of air filters or heparin. In this study, the overall incidence of DWI lesions was 11%. Interestingly, the incidence was 22% in the control group, but only 6% when air filters or heparin were used, respectively [9]. Hähnel et al. reported an incidence of 18.5% of asymptomatic lesions in a cohort of 22 patients undergoing diagnostic angiography and 5 patients undergoing an interventional procedure [12]. On the lower end of the spectrum of reported results, Britt et al. described an incidence of 0% in a study comprising 20 diagnostic angiographies [10]. This result, however, might be due to the narrow selection of patients. Using a Bayesian statistical approach, the authors predicted that cerebral angiography is associated with an incidence of asymptomatic cerebral infarction of no more than 9% (95% confidence).

To the best of our knowledge, our study comprises the largest patient cohort reported so far. In our study, we emphasized the importance of including a wide range of factors in the evaluation. Our main finding is that we found asymptomatic DWI lesions in 59 of 344 diagnostic examinations, corresponding to an incidence of 17%. As outlined above, it is not straightforward to compare our results to the literature, since diagnostic and interventional procedures have been grouped together in most of the previously published reports. Although our results are reasonably consistent with the literature, we had expected to find a lower incidence. Bendszus et al. were able to reduce their incidence of DWI lesions to 6% when heparin was administered as a bolus and as a continuous infusion. Since all our angiographies were performed using catheters continuously flushed with heparinized NaCl solutions, we expected a similar result. Possibly, an additional heparin bolus of 50 I.U./kg BW as used by Bendszus et al. is required to protect patients from ischemia. On the other hand, we did not obtain DWI prior to performing the angiographies. Thus, it cannot be ruled out that in some of our patients, DWI lesions were already present before angiography, and that consequently, the true incidence of DWI lesions caused by angiography in our cohort is somewhat lower than 17%. Nevertheless, our data indicate that although our center was able to achieve an incidence of permanent neurological damage after an angiography as low as 0.13% [13], the frequency of asymptomatic cerebral ischemia is considerably higher.

In our study, we also aimed to identify risk factors associated with the number and location of lesions. Predisposing risk factors present in our cohort included higher patient age, history of atherosclerosis, cerebral infarction, or chronic heart disease (CHD), increased fluoroscopy time, and increased amount of injected contrast medium. In the literature, many factors have been described to increase the risk during cerebral angiography. In this context, age is the most relevant factor [4,7,14]. Cardiovascular diseases [7,8,15,16], the number of catheters used [8,11], the amount of contrast medium [8,11], and fluoroscopy time [7,8] have also already been described to increase the risk of suffering from symptomatic ischemic lesions. So far, our results compare well with the literature.

DWI sequences have been established as the most sensitive tool for both reliable and early diagnosis of cerebral ischemia [17,18,19,20]. Using DWI sequences, we found 128 lesions that were 1–5 mm in size and only 39 lesions larger than 5 mm. The largest extent of a lesion was 10 mm. The review of studies shows that most lesions do not exceed the size between 1–5 mm, thereby confirming our results [3,8,11,12]. Nevertheless, any lesion, regardless of its size, is a sign of brain tissue damage [8,9]. We hypothesize that most embolic infarcts occur due to dislodged superficial microthrombi in the vessels caused by probing with catheters or guidewires. Other possible explanations include air emboli or foreign body emboli caused by contrast injections [12,21,22,23,24]. Consistent with our study, cortical and subcortical locations of lesions, as well as a location in areas previously probed, have been described most frequently [2,8,12,23].

In our study, it was remarkable that four patients had diffusion restrictions in areas not examined. In one patient, we found a prominent ramus communicans anterior, which, despite probing the right side, could have caused a lesion on the opposite side. Other explanations include the washout of thrombi from the aortic arch or cardiac arrhythmias that could have caused thrombus embolization of the contralateral brain tissue.

Limitations: We are aware that this study has limitations. Due to the retrospective data collection, a selection bias is present. It was not until 2018 that an attempt was made to systematically perform MRIs after every diagnostic angiography. Before 2018, MRIs were often requested only when they were required for differential diagnosis, or when patients showed changes in their clinical status. However, we carefully reviewed the medical documentation to ensure that this cohort included only patients who were asymptomatic for possible ischemias after angiography. In addition, there were no routine MRIs performed before angiography, with the result that possible preexisting infarcts were not detected. Thus, due to the design of our study, the causality of the angiographies for the DWI lesions cannot be proven. However, lesions were found in areas of non-probed vessels in only 4 patients, whereas 55 patients displayed DR only in areas that were supplied by arteries probed during angiography. In a second study, also including data from 2340 consecutive neuroangiographies performed over a period of 10 years, we analyzed all technical and clinical complications observed [13]. However, we deliberately excluded asymptomatic DWI lesions in that study because MRI was available only for a subset of patients. Given the different methodological approaches used and the divergent patient cohort, we decided to keep these two studies separate.

## 5. Conclusions

Our results indicate that although the risk of neurologic complications after diagnostic angiography for interventions performed in specialized neuroradiologic centers is low, asymptomatic cerebral ischemias are still comparatively frequent. Consequently, there is a need to further increase the safety of angiography procedures. Especially in elderly patients with cardiovascular and other risk factors as listed above, alternative methods should be considered. Studies with acetylsalicylic acid, air filters, or heparinization have already shown that a reduction in the number of silent ischemias during angiography is possible [9,24]. Refinement of angiographic methodology may also have an impact on the frequency of foreign body embolism [25].

## Figures and Tables

**Table 1:** Analysis of patient characteristics. Comparison of relevant factors in 285 patients without DWI lesions and 59 patients with DWI lesions. * Significant *p*-values.

Factor	No DWI Lesions [*n* = 285]	DWI Lesions [*n* = 59]	*p*-Value
Demographics:			
Age, years (median, IQR, range)	55, IQR: 20, 0–84	61, IQR: 14, 40–82	<0.001 *
Female sex, *n* (%)	162 (56.8%)	33 (55.9%)	0.898
**Indications:**			
Hemorrhage, *n* (%)	94 (32.2%)	24 (40.7%)	0.257
Vasculitis, *n* (%)	9 (3.1%)	3 (5.1%)	0.463
Tumor, *n* (%)	4 (1.4%)	0 (0%)	0.360
Stenosis, *n* (%)	34 (11.9%)	8 (13.6%)	0.728
Aneurysm, *n* (%)	21 (7.3%)	3 (5.1%)	0.531
Vascular anomaly, *n* (%)	40 (14.0%)	8 (13.6%)	0.924
Postoperative control, *n* (%)	22 (7.7%)	3 (5.1%)	0.478
Progress control, *n* (%)	56 (19.6%)	9 (15.3%)	0.433
Other, *n* (%)	5 (1.7%)	1 (1.7%)	0.975
**Risk factors:**			
Atherosclerosis, *n* (%)	17 (6.0%)	9 (15.3%)	0.014 *
Vasculitis, *n* (%)	3 (1.1%)	1 (1.7%)	0.675
Diabetes mellitus, *n* (%)	31 (10.8%)	8 (13.6%)	0.554
Hypertension, *n* (%)	143 (50.1%)	37 (62.7%)	0.079
Hereditary vascular disease, *n* (%)	4 (1.4%)	1 (1.7%)	0.865
Pulmonary embolism, *n* (%)	10 (3.5%)	2 (3.4%)	0.964
Atrial fibrillation, *n* (%)	8 (2.8%)	2 (3.4%)	0.812
Persistent foramen ovale, *n* (%)	3 (1.1%)	0 (0%)	0.429
Coagulation abnormalities, *n* (%)	8 (2.8%)	2 (3.4%)	0.808
Cerebral infarction, *n* (%)	32 (11.2%)	13 (22%)	0.026 *
Heart attack/			
coronary heart disease (CHD), *n* (%)	10 (3.5%)	6 (10.2%)	0.027 *
Deep vein thrombosis, *n* (%)	7 (2.52%)	1 (1.7%)	0.724
Peripheral arterial disease, *n* (%)	4 (1.4%)	0 (0%)	0.360

**Table 2:** Details of DSA procedures. Comparison of relevant factors in 285 patients without DWI lesions and 59 patients with DWI lesions. * Significant *p*-values.

Factor	No DWI Lesions [*n* = 285]	DWI Lesions [*n* = 59]	*p*-Value
**No. of catheters used:**			0.066
1	231 (81.1%)	42 (71.2%)	
>1 [max. 7]	54 (18.9%)	17 (28.8%)	
**Angiographic approach:**			0.255
Femoral right, *n* (%)	257 (90.2%)	56 (94.9%)	
Femoral left, *n* (%)	22 (7.7%)	2 (3.4%)	
Femoral both sides, *n* (%)	3 (1.1%)	0 (0%)	
Brachial, *n* (%)	1 (0.4%)	0 (0%)	
Radial right, *n* (%)	1 (0.4%)	0 (0%)	
Radial left, *n* (%)	1 (0.4%)	0 (0%)	
Direct carotid, *n* (%)	0 (0%)	1 (1.7%)	
No. of probed vessels, median (range)	5 (1–25)	5 (1–8)	0.0980
Probing time [minutes], median (IQR)	30, IQR: 31.5	39, IQR: 36.3	0.290
Fluoroscopy time [minutes], median (IQR)	9.4, IQR: 8.7	12, IQR: 9.9	0.033 *
Radiation exposure [µGym^2^], median (IQR)	6054.7, IQR: 6726.5	6685, IQR: 6742.5	0.266
Contrast material [ml], median (IQR)	100, IQR: 100	150, IQR: 100	0.047 *
Weekend shift or night shift, *n* (%)	45 (15.8%)	9 (15.3%)	0.918
1.5 T, *n* (%)	125 (43.9%)	26 (44.1%)	0.977
3 T, *n* (%)	160 (56.1%)	33 (55.9%)	0.977

## Data Availability

Data supporting reported results can be found (links will be provided during review).
